# College and Hospital Note

**Published:** 1908-09

**Authors:** 


### COLLEGE AND HOSPITAL NOTE

---

THE Medical Department of the University of Buffalo will begin its Sixty-third annual session, Monday, September 28, 1908. The opening lecture will be delivered by Dr. Julius Ullman, instructor in clinical medicine, at Alumni Hall in the University building, at 8 o'clock p. m. A general invitation to the profession to attend is extended by the faculty.

